# Early life nutrition affects the molecular ontogeny of testicular development in the young bull calf

**DOI:** 10.1038/s41598-022-23743-3

**Published:** 2023-04-25

**Authors:** Stephen Coen, Kate Keogh, Pat Lonergan, Sean Fair, David A. Kenny

**Affiliations:** 1 Teagasc Animal and Grassland Research and Innovation Centre, Grange, Dunsany, Co. Meath Ireland; 2 School of Agriculture and Food Science, University College Dublin, Belfield, Dublin 4, Ireland; 3 Laboratory of Animal Reproduction, Department of Biological Sciences, University of Limerick, Limerick, Ireland

**Keywords:** Molecular biology, Physiology

## Abstract

Enhanced early life nutrition accelerates sexual development in the bull calf through neuroendocrine-signalling mediated via the hypothalamic–pituitary–testicular axis. Our aim was to assess the impact of contrasting feeding regimes in bull calves during the first 12 weeks of life on the testes transcriptome and proteome. Holstein–Friesian bull calves were offered either a high (HI) or moderate (MOD) plane of nutrition, designed to support target growth rates of 1.0 and 0.5 kg/day, respectively. At 12 weeks of age all calves were euthanized, testicular parenchyma sampled, and global transcriptome (miRNAseq and mRNAseq) and proteome analyses undertaken. Bioinformatic analyses revealed 7 differentially expressed (DE) miRNA and 20 DE mRNA. There were no differentially abundant proteins between the two dietary groups. Integration of omics results highlighted a potential role for the cadherin gene, *CDH13*, in earlier reproductive development. Furthermore, co-regulatory network analysis of the proteomic data revealed CDH13 as a hub protein within a network enriched for processes related to insulin, IGF-1, androgen and Sertoli cell junction signalling pathways as well as cholesterol biosynthesis. Overall, results highlight a potential role for CDH13 in mediating earlier reproductive development as a consequence of enhanced early life nutrition in the bull calf.

## Introduction

The advent of genomic selection has intensified the demand for saleable semen from genetically elite dairy bulls at an increasingly younger age. Dairy bred bulls can reach puberty from 8 to 11 months^1^; however, this is dependent on management practises. Enhanced early life plane of nutrition is known to accelerate sexual development in the bull calf^2–4^. We^5^ and others^6^ have shown that this is mediated through complex biochemical interplay between metabolic cues and neuroendocrine signalling within the hypothalamic–pituitary–testicular signalling axis, leading to precocious testicular development, testosterone production and spermatogenesis. Indeed, Dance, et al.^6^ reported that calves offered an enhanced plane of nutrition from 2 to 32 weeks of age reached puberty earlier, had larger testes and increased sperm production potential than those on a lower plane at 32 weeks of age^6^. In a similar study from our own group^7^, greater testicular histological development was observed through for example, advanced stage of spermatogenesis and greater Sertoli cell number in calves offered an enhanced diet compared to those offered a lower plane of nutrition up to 18 weeks of age. This effect of enhanced early life dietary intake on subsequent reproductive development is due to an earlier transient rise in systemic concentrations of LH which typically occurs between 8 and 20 weeks of age, with a peak at 12–15 weeks, after which it begins to decline at approximately 25 weeks^8^. We have clearly shown the impact of early life nutrition on LH secretion, whereby retardation in sexual development and age at puberty onset as a consequence of poor dietary management during the first 6 months of life is non compensable, even if unrestricted access to a high energy diet is offered thereafter^5^. In order to more precisely uncover the effect of metabolic status on aspects of the physiological and molecular regulation of reproductive and metabolic development, it is important to gain a better understanding of an earlier period of development (up to 12 weeks of age) which coincides with the start of the approximate peak of the aforementioned LH transient rise.

While we^7^ and others^9^ have investigated aspects of the impact of early life nutrition on global mRNA transcriptomic profiles of testicular tissue, an evaluation of both miRNA transcriptomic and global proteomics in relation to this phenotype is lacking. Therefore, the objective of this study was to examine the effect of enhanced nutritional status during early calfhood on (i) the global testicular transcriptome (miRNA and mRNA) and (ii) the testicular proteome in bull calves coincident with the early transient gonadotrophin rise (12 weeks). Moreover, a more in-depth integrative approach is warranted in order to better elicit the key transcript and protein networks underlying observed phenotypic variance as well as potentially elucidating cause and effect relationships. Thus, we also aimed to integrate results from the global transcriptome and proteome analyses in order to highlight differentially expressed (DE) miRNA regulating DE genes as well as determining the interaction amongst proteins identified within the testes parenchyma tissue.

## Materials and methods

This study was conducted at the Teagasc Animal and Grassland Research and Innovation Centre. All procedures involving animals were approved by the Teagasc Animal Ethics Committee and licensed by the Irish Health Products Regulatory Authority in accordance with the European Union Directive 2010/63/EU. All experiments were performed in accordance with relevant regulations and the ARRIVE (Animal Research: Reporting on In Vivo Experiments) guideline.

### Experimental design and animal management

The animal model and general management regimen pertaining to the animals used in this study have previously been extensively described^10^. Briefly, thirty Holstein–Friesian bull calves with a mean (± SD) age and bodyweight of 17.5 (± 2.85) days and 48.8 (± 5.3) kg, respectively, were sourced from four commercial dairy farms and blocked on age, sire, liveweight and farm of origin. The experimental animal model utilised a randomised complete block design, and all animals were balanced across 4 pens with regards to treatment, age, farm of origin, weight and sire (17 sires across 30 calves with a max of 3 calves per sire). In total there were 4 blocks of calves, with block represented across the 4 housing pens used for the study. After seven days of acclimatisation, calves were assigned to either a high (HI) or moderate (MOD) plane of nutrition. Calves were individually fed milk replacer and pelleted concentrate using an electronic calf feeding system (Forster-Tecknik Vario; Engen, Germany). Calves on the HI received 1500 g of milk replacer in 10 L of water daily, together with concentrate *ad-libitum*. Calves on MOD were allocated 500 g of milk replacer in 4 L of water plus a maximum of 0.5 kg of concentrates daily. All calves had daily access to fresh water and approximately 0.5 kg of hay. At a mean age (± SD) of 87 (± 2.14) days, all calves were euthanized following intravenous administration of an overdose of sodium pentobarbitone (300 mg/ml: 0.25 mL/kg bodyweight). The testes were excised from each calf and the tunica albuginea, epididymides and any excess connective tissue removed. The testes parenchyma tissue was harvested avoiding the rete testis and snap-frozen in liquid nitrogen and subsequently stored at − 80 °C pending further processing.

### Global proteomics analysis

#### RNA isolation

Total RNA was isolated from all testes samples (n = 30) using the Qiagen RNeasy Plus Universal Mini kit (Qiagen Ltd, Manchester, UK) according to the manufacturers instructions including steps for the purification of Total RNA containing miRNA. Yield of the resultant RNA was determined by measuring the absorbance at 260 nm with a Nanodrop spectrophotometer (NanoDrop Technologies; Wilmington, DE, USA). The quality of the isolated RNA was evaluated on an Agilent 2100 Bioanalyser (Agilent Technologies Ireland Ltd., Dublin, Ireland). All samples were deemed to be of excellent quality for subsequent RNA sequencing, with an overall average RNA Integrity Number of 9.5.

#### RNAseq library preparation and sequencing

Both mRNA and miRNAseq library preparation and sequencing were undertaken by a commercial sequencing facility (Macrogen Europe Inc., Amsterdam, The Netherlands). Individual cDNA libraries were prepared from all 30 total RNA samples using the Illumina Truseq stranded mRNA kit for mRNAseq and the Illumina small RNA kit for miRNA, according to the kit instructions. High-throughput sequencing was undertaken on an Illumina NovaSeq, incorporating 150 bp paired end sequencing for mRNA and an Illumina HiSeq 2500 sequencer, incorporating 50 bp single end sequencing for small RNA sequencing.

#### miRNAseq and mRNAseq bioinformatic analyses

Both mRNA and miRNA raw sequence reads were firstly assessed for sequencing quality using FASTQC (version 0.11.8, https://www.bioinformatics.babraham.ac.uk/projects/fastqc/). The Illumina sequencing adaptor was clipped off all the raw read sequences using Cutadapt (version 1.18). For the miRNA data, reads of lengths shorter than 15 bp, and longer than 28 bp were subsequently removed as short and long reads, respectively, using Cutadapt. The retained reads were then additionally filtered for other bovine short RNA species including ribosomal RNAs (rRNAs), transfer RNAs (tRNAs), small nuclear RNAs (snRNAs) and small nucleolar RNAs (snoRNAs) downloaded from https://rnacentral.org/. To profile miRNA expression in each sample, the miRDeep2 package (version 2.00.8) modules were used, together with the bovine reference genome (ARS-UCD1.2) and the known bovine mature miRNA sequences and their precursor sequences from the miRBase database (release 22.1). The miRDeep2 mapper module (mapper.pl) was then used with default parameters to collapse reads of the sequences into clusters. Bowtie (version 1.1.1) was then employed to align the collapsed reads to the indexed reference genome. Using default parameters and input files including the reference genome, collapsed reads versus reference genome alignment, known bovine mature miRNAs and their precursors sequences (including the hairpin structures) and *Bos taurus* (bta) as the species of interest, the miRDeep2 module (miRDeep2.pl) was used to quantify bovine miRNAs. Through this, the miRDeep2 quantifier module was used to quantify all known expressed miRNAs in the sequence data, producing read counts for each individual sample. For the mRNA sequencing data, the Spliced Transcripts Alignment to a Reference (STAR) aligner^11^ was employed to align sequencing reads to the ARS-1.2 version of the bovine reference genome. Additionally, within the STAR alignment script, the quantmode function was included in order to quantify the number of reads aligned to each gene. Resultant read counts for each sample across each mRNA and miRNA datasets were then merged into two separate files (miRNA reads and mRNA reads) and subsequently assessed separately for differentially expressed miRNA and mRNA using the R (v2.14.1) Bioconductor package, edgeR (version 3.26.7). For this genes read counts were firstly filtered for the removal of lowly expressed genes, whereby any gene with less than one count per million in at least half the number of samples (n = 15) was removed from the analysis. Retained read counts were then normalised using the trimmed mean of M-values normalisation method. Normalized read counts were then analysed with a generalized linear model, incorporating block within the model, however block was found to not be significant. Genes with a Benjamini–Hochberg false discovery rate of 10% and a fold-change greater than 1.5 were considered differentially expressed. Target genes for differentially expressed miRNAs were predicted using TargetScan (release 7.2; http://www.targetscan.org/vert_72/).

### Global proteomics analysis

#### Sample preparation

The methodology has been previously described in^12^. Global proteomics analysis was undertaken on the same tissue samples as used for RNAseq. For each sample, proteins were extracted using a tissue homogenizer (TissueLyser II, Qiagen) and digested using a commercial iST Kit (PreOmics, Germany). Briefly, 50 µl of ‘Lyse’ buffer and around 50 ug of glass beads (425–500 µm, Sigma) were added to the thawed tissue. After 2 cycles of protein extraction (2 min each, 30 Hz) and 10 min at 95 °C, the tubes were centrifuged at 16,100×*g* for 15 min, and the supernatant was pipetted into a fresh Eppendorf tube for proteomics analysis. The solubilization of the extracted proteins was enhanced by subjecting the samples with High Intensity Focused Ultrasound (HIFU) for 1 min setting the ultrasonic amplitude to 85%. The protein concentration was estimated using the Qubit^®^ Protein Assay Kit (Life Technologies, Zurich, Switzerland). For each sample, 50 µg of protein were transferred to the cartridge and digested by adding 50 µl of the ‘Digest’ solution. After 60 min of incubation at 37 °C the digestion was stopped with 100 µl of ‘Stop’ solution. The solutions in the cartridge were removed by centrifugation at 3,800 g, while the peptides were retained by the iST-filter. Finally, the peptides were washed, eluted, dried and re-solubilized in 20 µL of injection buffer (3% acetonitrile, 0.1% formic acid).

#### Liquid chromatography–mass spectrometry analysis

Mass spectrometry (MS) analysis was performed on a Q Exactive HF-X mass spectrometer (Thermo Scientific) equipped with a Digital PicoView source (New Objective) and coupled to a M-Class UPLC (Waters)^12^. Solvent composition at the two channels was 0.1% formic acid for channel A and 0.1% formic acid, 99.9% acetonitrile for channel B. For each sample, 1 μL of peptides was loaded on a commercial MZ Symmetry C18 Trap Column (100 Å, 5 µm, 180 µm × 20 mm, Waters) followed by nanoEase MZ C18 HSS T3 Column (100 Å, 1.8 µm, 75 µm × 250 mm, Waters). The peptides were eluted at a flow rate of 300 nL/min by a gradient from 8 to 27% B in 85 min, 35% B in 5 min and 80% B in 1 min. Samples were acquired in a randomized order. The mass spectrometer was operated in data-dependent mode (DDA), acquiring a full-scan MS spectra (350–1′400 m/z) at a resolution of 120′000 at 200 m/z after accumulation to a target value of 3,000,000, followed by HCD (higher-energy collision dissociation) fragmentation on the twenty most intense signals per cycle. HCD spectra were acquired at a resolution of 15′000 using a normalized collision energy of 25 and a maximum injection time of 22 ms. The automatic gain control (AGC) was set to 100′000 ions. Charge state screening was enabled. Singly, unassigned, and charge states higher than seven were rejected. Only precursors with intensity above 250,000 were selected for MS/MS. Precursor masses previously selected for MS/MS measurement were excluded from further selection for 30 s, and the exclusion window was set at 10 ppm. The samples were acquired using internal lock mass calibration on m/z 371.1012 and 445.1200.

#### Protein identification and label free protein quantification

The acquired raw MS data were processed by MaxQuant (version 1.6.2.3), followed by protein identification using the integrated Andromeda search engine^13^. Spectra were searched against a Uniprot *Bos taurus* reference proteome (taxonomy 9913, version from 2017-08-17), concatenated to its reversed decoyed fasta database and common protein contaminants. Carbamidomethylation of cysteine was set as fixed modification, while methionine oxidation and N-terminal protein acetylation were set as variable. Enzyme specificity was set to trypsin/P allowing a minimal peptide length of 7 amino acids and a maximum of two missed cleavages. MaxQuant Orbitrap default search settings were used. The maximum false discovery rate (FDR) was set to 0.01 for peptides and 0.05 for proteins. Label free quantification was enabled and a 2-min window for match between runs was applied. In the MaxQuant experimental design template, each file is kept separate in the experimental design to obtain individual quantitative values. Protein fold changes were computed based on Intensity values reported in the proteinGroups.txt file. Statistical analysis between treatment groups was conducted using Perseus V.1.5.8.5 (http://www.maxquant.org). A set of functions implemented in the R package SRMService^14^ was used to filter for proteins with 2 or more peptides allowing for a maximum of 10 missing values, and to normalize the data with a modified robust z-score transformation and to compute p-values using the t-test with pooled variance. Label-free quantitative intensities were Log2-transformed and ANOVA of significance, incorporating block in the model was undertaken to determine proteins differentially abundant between the two dietary treatment groups examined.

### Integration of miRNA, mRNA and proteomic results

In order to evaluate the relationship between miRNA, mRNA and proteins, the various ‘omics’ datasets pertaining to the current study were integrated. This was achieved by firstly determining a relationship between the miRNA and mRNA results; specifically, the mRNA targets of the DE miRNA were assessed for the presence of DE mRNA genes. A weighted co-regulatory network analysis was then undertaken on the proteomics data and resultant networks mined for DE genes (which were affected/targeted by DE miRNA) using the WGCNA R software program^15^. Label-free quantitation intensity values were Log_2_ transformed in R. The WGCNA automatic network construction and module detection method was utilised to generate unsigned co-regulated networks. For each separate analysis, pair-wise weighted Pearson correlations were calculated between all pairs of proteins in the testes dataset. Adjacency matrices were calculated to reach scale-free topology of the network (R^2^ > 0.09) by raising the co-regulation matrix to a soft-threshold power of 14. Following this, the topology overlap matrix was calculated, providing information on the similarity of the co-regulation between two proteins with all other proteins in the network. Average linkage hierarchical clustering was then applied to the topology overlap matrix resulting in the grouping of modules of co-regulated proteins. Resulting modules or networks of co-expressed proteins were mined for DE mRNA genes affected by DE miRNAs to determine the interactions of these transcripts with the testicular proteome as affected by prevailing early life nutrition.

### Gene and protein pathways analysis

In order to determine biological pathways and functions enriched by the dietary treatments imposed, miRNA target genes, DE mRNA genes and protein network analysis results were individually subjected to pathway analysis using Ingenuity Pathway Analysis (IPA) according to the manufacturer’s instructions (Qiagen Inc., https://www.qiagenbioinformatics.com/products/ingenuitypathway-analysis^15^.

## Results

### Animal model

The effect of the two contrasting dietary regimens imposed on calf growth rate and key metabolic and reproductive endocrinological responses have been described previously in detail^10^ and for context is briefly summarised here. Average daily gain (ADG) was 35% higher for calves on the HI compared with the MOD group. At slaughter, calves were 23.7 kg heavier in the HI compared with the MOD (112.4 v 88.7 (2.98) kg, P < 0.001) group and this growth advantage was consistently reflected throughout the experimental period with HI calves having a greater ADG (0.88 v 0.58 kg, P < 0.001, respectively). Paired testicular weight at slaughter was also higher (29.2 v 20.1 (2.21) g; P = 0.0003) for calves on the HI compared to the MOD plane of nutrition.

### miRNAseq analyses

miRNA sequencing resulted in the generation of an average of 13.8 million reads per sample, with an associated alignment rate of 89.2% on average. Global miRNA analysis in this study identified seven miRNAs as differentially expressed (adj. P-value < 0.1; fold change > 1.5) between the HI and MOD dietary treatment groups (Table 1). miR-11995, miR-146b, miR-34b, miR-34c and miR-2419-5p were up-regulated, with miR-223 and miR-10a being down-regulated in the testes of calves on the HI versus MOD diet. Additionally, TargetScan analysis revealed putative target mRNAs of the DE-miRNA, pathway analysis results for the DE miRNA targets included enrichment of processes involved in Sertoli cell-Sertoli cell junction signalling, formation and cell viability of Sertoli cells, androgen signalling and AMPK signalling. Enriched pathways for the various miRNA are presented in Supplementary Table 1. Raw sequencing reads and gene counts for each sample utilised in this study have been deposited within NCBI’s Gene Expression Omnibus and are available through GEO ID GSE194041.

**Table 1:** Differentially expressed miRNA in the testes of Holstein–Friesian dairy bull calves fed differentially up to 12 weeks of age.

MiRNA	logFC^a^	Adj. p-value
bta-miR-223	− 0.99385	0.04786
bta-miR-10a	− 0.6483	0.024534
bta-miR-34b	0.649572	0.033159
bta-miR-34c	0.731756	0.008514
bta-miR-2419-5p	0.736173	0.016119
bta-miR-11995	0.753923	0.012793
bta-miR-146b	1.218274	0.006569

^a^Positive logFC value indicates up-regulation and negative indicates down-regulation in the HI group compared to the MOD group.

### mRNAseq analyses

**Figure 1:**
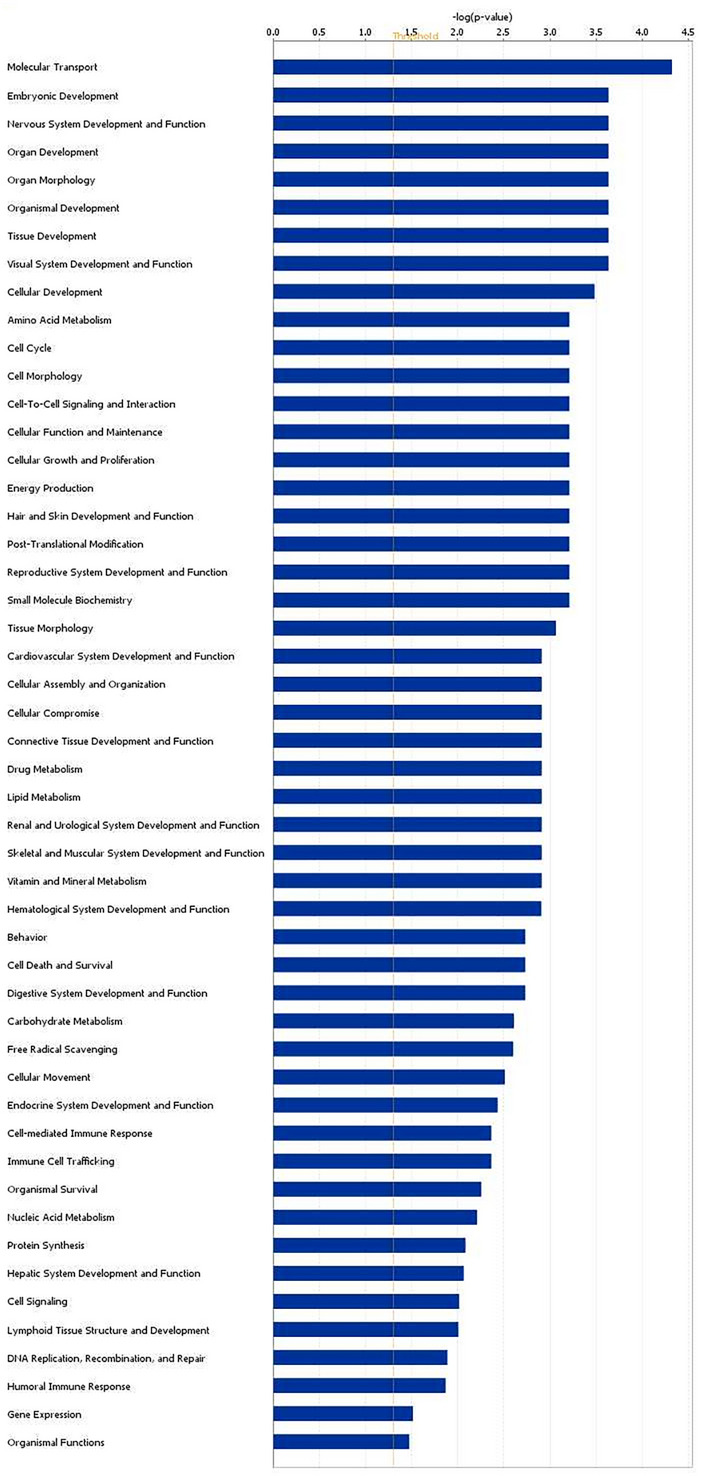
Top functions of genes differentially expressed in bull testes at 12 weeks as per Ingenuity Pathway Analysis (IPA) (Top 25 functions shown). Functions are marked on the X-axis and -log (p value) marked on the Y-axis. Right-tailed Fisher’s exact test was used to calculate the p-value determining the probability that each biological function assigned to that data set is due to chance alone (P < 0.05). This image was generated through the use of IPA (QIAGEN Inc., https://www.qiagenbio-informatics.com/products/ingenuity-pathway-analysis)^15^’.

**Figure 2:**
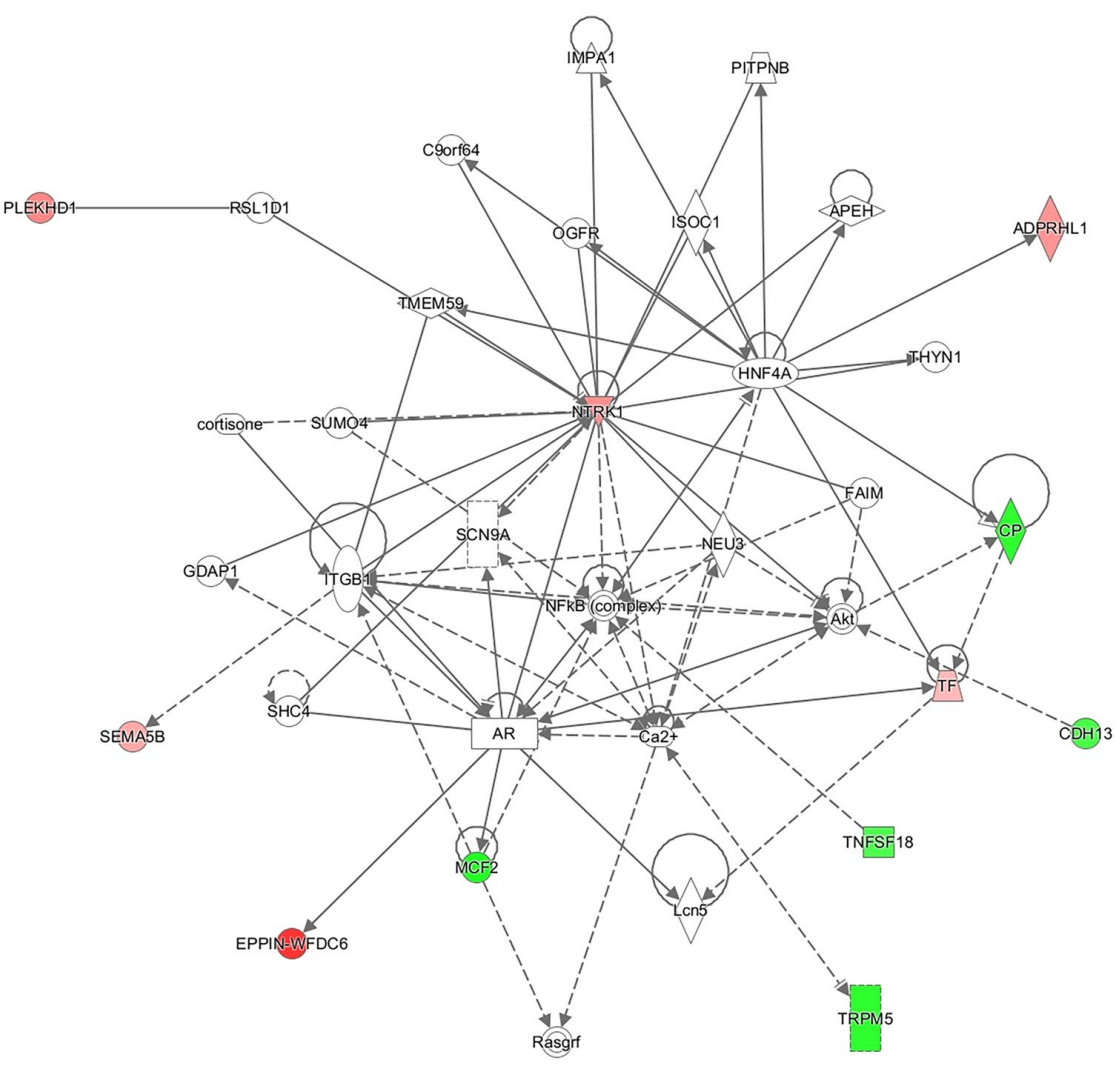
Network involving genes expressed in testes tissue of bull calves two divergent planes of nutrition for the first 12 weeks of life. The network is displayed graphically as nodes (genes). The node colour intensity indicates the expression of genes, with green representing down-regulation in calves fed the HI plane of nutrition compared to those fed a MOD plane of nutrition. Pink/Red represents up-regulation in HI compared with MOD calves. This image was generated through the use of IPA (QIAGEN Inc., https://www.qiagenbio-informatics.com/products/ingenuity-pathway-analysis)^15^’.

An average of 65 million reads were generated across each sample through mRNA sequencing. Alignment of trimmed sequencing reads to the bovine genome resulted in an average mapping rate of 86%. EdgeR analysis resulted in the identification of 20 differentially expressed genes (adj. P-value < 0.1; fold change > 1.5) between treatment groups (Table 2). Functional analysis of DE genes revealed enrichment of processes related to amino acid metabolism, lipid metabolism, energy production and cellular growth and proliferation and reproductive system development and lipid metabolism (Fig. 1; Supplementary Table 2). Biological processes related to reproductive system development included entry into meiosis of male germ cells; cell viability of Sertoli cells and formation of Sertoli cells, established through differential expression of *CYP26B1, TF,* and *NTRK1*, respectively. Moreover, through IPA, networks based on DE genes were derived (Supplementary Table 3); network 1 (Fig. 2) was of particular interest due to its inclusion of genes involved in lipid metabolism and small molecule biochemistry. Raw sequencing reads and gene counts for each sample utilised in this study have been deposited within NCBI’s Gene Expression Omnibus and are available through GEO ID GSE194305.

**Table 2:** Differentially expressed mRNA in the testes of Holstein–Friesian dairy bull calves fed differentially up to 12 weeks of age.

GENE ID	Gene symbol	logFC^a^	Adj. p-value
ENSBTAG00000001219		− 1.73884	0.028289
ENSBTAG00000004007	*MCF2*	− 1.05748	0.041005
ENSBTAG00000037937		− 0.99015	0.041856
ENSBTAG00000046662	*TRPM5*	− 0.95831	0.011205
ENSBTAG00000012164	*CP*	− 0.95325	0.07947
ENSBTAG00000048448		− 0.84643	0.046812
ENSBTAG00000034373	*CDH13*	− 0.78502	0.039497
ENSBTAG00000048659		− 0.72665	0.018212
ENSBTAG00000047412	*TNFSF18*	− 0.72127	0.047498
ENSBTAG00000007273	*TF*	0.600004	0.07947
ENSBTAG00000012212	*CYP26B1*	0.683043	0.074
ENSBTAG00000006276	*C1QTNF1*	0.735242	0.039497
ENSBTAG00000018765	*SEMA5B*	0.904539	0.07947
ENSBTAG00000048655	*NT5E*	0.942715	0.07947
ENSBTAG00000012036	*HR*	1.013813	0.014565
ENSBTAG00000020928	*ADPRHL1*	1.204497	0.024944
ENSBTAG00000003060	*NTRK1*	1.283853	0.080685
ENSBTAG00000008576	*PLEKHD1*	1.388086	0.005261
ENSBTAG00000001495	*SPINLW1*	2.156874	0.011205
ENSBTAG00000040367		3.451802	0.011205

^a^Positive logFC value indicates up-regulation and negative indicates down-regulation in the HI group compared to the MOD group.

### Global proteomics analysis

**Figure 3:**
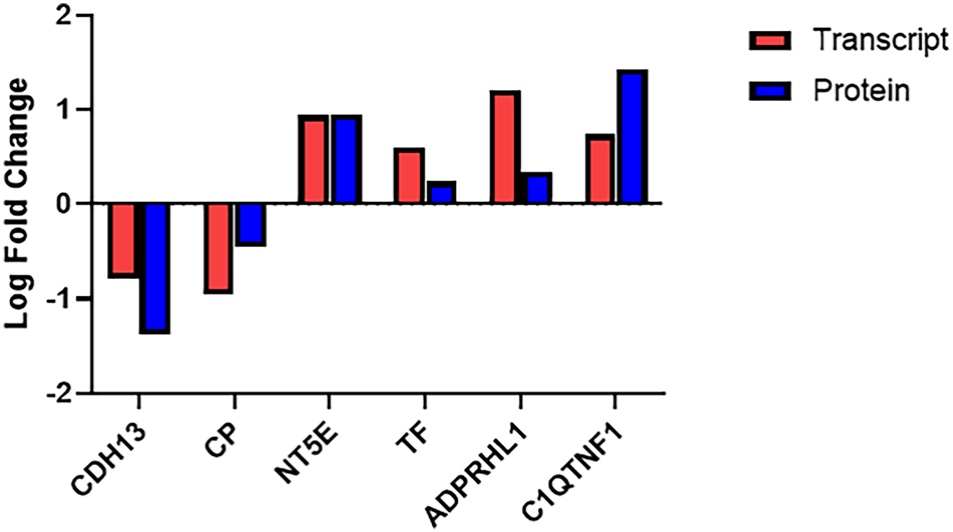
Comparison of expression between DE-mRNA transcripts identified within the proteomic dataset of the testes parenchyma.

Protein quantification and identification resulted in the identification of 5,665 proteins across the testes samples examined. The total number of proteins represents those with at least two peptides. Following implementation of correction for multiple testing (adj. P-value 0.1; fold change > 1.5), no protein was deemed to have reached statistical significance in terms of differential abundance between the two dietary groups. However, despite no proteins identified within the testes passing significance for multiple testing, corresponding proteins of DEGs were significant at an uncorrected p-value. For example, the following proteins were differentially abundant at an uncorrected p-value: C1QTNF1 (p = 0.00747); CDH13 (p = 0.00349); CP (p = 0.0257); NT5E (p = 0.00373). Additionally, corresponding proteins of other DE mRNAs including ADPRHL1 and TF were also identified within the proteomic dataset, with all proteins following the same direction of effect as the transcript data (Fig. 3). Proteomics data generated in this analysis have been uploaded to the ProteomeXchange Consortium via the PRIDE (http://www.ebi.ac.uk/pride) partner repository^16^ with the data identifier; PXD031403.

### Integrative network analyses

Based on the DE miRNA and DE mRNA results, a relationship was established between two miRNA, miR-2419-5P and miR-11995, as well as their putative target mRNA, *CDH13* and *TNFSF18*, which were differentially expressed between the HI and MOD dietary groups. Specifically, the two miRNAs were up-regulated in the HI calves, and their corresponding target mRNA were down-regulated in the same dietary group, suggesting mRNA suppression due to each respective miRNA. Co-regulatory network analysis of the testes proteome dataset resulted in the formation of 13 separate networks of co-regulated proteins, each denoted by an individual colour identifier. Proteins included within each network are presented in Supplementary Table 4. Of particular interest was the turquoise network which contained CDH13 as a hub protein. Pathway analysis of proteins directly interacting with CDH13 included those involved in cholesterol biosynthesis (Super pathway of Cholesterol Biosynthesis, Cholesterol Biosynthesis I, Cholesterol Biosynthesis II (via 24,25-dihydrolanosterol), Cholesterol Biosynthesis III (via Desmosterol)), growth hormone signalling and those involved in Sertoli cell development (Germ Cell-Sertoli Cell Junction Signalling, Sertoli Cell-Sertoli Cell Junction Signalling), presented in Supplementary Table 5.

## Discussion

Genes differentially expressed in this study revealed an enrichment of biological processes and pathways related to metabolic status and reproductive development. However, despite differences at the transcript level, no protein was identified as significantly differentially abundant between treatment groups when based on a corrected p-value. This lack of significant differences within the proteomic dataset compared to the transcriptomic dataset may be due to differences in analytical techniques and sensitivities between the two analytical methods, namely next generation sequencing and mass spectrometry, which was evident through the large difference in the numbers of protein-coding transcripts and proteins identified between the two analyses. However, despite not identifying any proteins as significantly differentially abundance, through our secondary aim of determining the interaction between proteins within the testes parenchyma, 13 separate networks of co-regulated proteins were derived. Moreover, integration of DE-miRNA, DE-mRNA with networks of co-regulated proteins from the same individuals revealed the possible regulatory roles of specific miRNAs on protein abundance in testicular development as a consequence of augmented dietary intake, during early life.

Augmented early-life plane of nutrition resulted in the differential expression of seven miRNA; moreover, these DE miRNAs potentially targeted a diverse range of mRNA transcripts, apparent through the enrichment of various biological pathways and functions. Indeed, despite the varied enrichment of biological processes across the 7 DE miRNA, commonality of target mRNA was also apparent. For example, pathways that were commonly enriched based on miRNA target genes included IGF-1 signalling, androgen signalling and Sertoli cell-Sertoli cell junction signalling. Moreover, specific miRNA identified as DE were previously implicated in testicular or reproductive development. Specifically, Gao et al.^17^ reported a significant role for miR-146b in the regulation of bovine testis development manifested as greater expression in adult compared to neonatal testes tissue. Moreover, over expression of miR-34c promoted the expression of genes involved in meiosis in mouse spermatogenesis^18^, whilst Xu et al.^19^ reported that down-regulation of miR-34b/c cluster in cattleyak (Hybrid of cattle and Yak (*Bos grunniens*)) potentially contributed to the failure of the transition from mitosis to meiosis and subsequent spermiogenesis. Additionally, another study suggests that transgenic constructs of miR-34b/c are essential for normal spermatogenesis and male fertility in mice, but their presence in sperm is dispensable for fertilization and preimplantation development^20^. Furthermore, miR-34c expression was also observed to be altered in underfed adult rams, and these variations were postulated to cause reduction in spermatozoa quality by disruption of Sertoli cell function and an increase germ cell apoptosis^21^. As the aforementioned genes were all up-regulated in the current study, this may suggest that the transition from mitosis to meiosis and subsequent spermiogenesis was more advanced in calves that received the HI plane of nutrition. A similar study from our group^22^, reported advanced spermiogenesis at 18 weeks of age in calves on a high compared to a moderate plane of nutrition, consistent with the results of the current study. Another miRNA up-regulated in the HI group was miR-2419-5p; pathway analysis of target genes of this miRNA included enriched pathways such as Sertoli cell-Sertoli cell junction signalling, AMPK signalling and IGF-1 signalling. AMPK functions in metabolic coordination^23^; thus, enrichment of this pathway may suggest activation of AMPK due to differential feeding in early life. Moreover, a greater requirement for metabolic coordination consistent with increased dietary intake may allow more growth potential, evident through enrichment of IGF-1 signalling, with greater growth then leading to earlier Sertoli cell development. Similarly, miR-11995 was also up-regulated in the HI calves, with target genes enriched in biological pathways including AMPK Signalling as well as Androgen signalling, suggesting a role for miR-11995 towards mediating enhanced metabolic status with testicular development in the HI calves.

In addition to DE miRNAs, we also observed differential expression of 18 protein coding genes. Pathway analysis revealed enrichment of biological processes related to reproductive system development, apparent through the differential expression of *CYP26B1, TF,* and *NTRK1*, which have roles in entry into meiosis of male germ cells (*CYP26B1*), cell viability of Sertoli cells (*TF)* and formation of Sertoli cells (*NTRK1*). Indeed, the three mentioned genes were all up-regulated in the HI calves, indicating earlier reproductive development in those calves. This is further established through upstream analysis using IPA, where testosterone was predicted to regulate the expression of *CYPB26B1, NTRK1* and *TF.* Indeed, systemic concentrations of testosterone were greater in the HI calves in the current study^10^. Moreover, a relationship between the three aforementioned genes and testicular development in early life was recently established through co-expression network analysis, whereby a network containing *CYPB26B1, NTRK1* and *TF* which was positively associated with greater Sertoli cell number, more advanced stage of spermatogenesis and greater concentrations of testosterone in the testes of bull calves fed an enhanced diet up to 18 weeks of age^24^. *CYP26B1* is involved in retinoic acid (RA) catabolism contributing to the maintenance of testis development^25–27^. Moreover, *NTRK1* and *TF* were also included within Network 1 (Fig. 2) derived through IPA and depicted genes involved in cellular movement, lipid metabolism and small molecular biochemistry. Furthermore, other differentially expressed genes within this network were previously implicated in testicular development following enhanced early life plane of nutrition up to 18 weeks of age (*TF, NTRK1, SEMA5B*)^7^ as well as both 16 and 24 weeks of age (*TF, MCF2*, *PLEKHD1*)^9^. The genes listed above (*TF, NTRK1, SEMA5B, MCF2* and *PLEKHD1*) that were also differentially expressed in similar studies play important roles in testicular, spermatogenic and overall sexual development. The *NTRK1* gene, encodes a receptor for nerve growth factor^28,29^; and Niu^30^, reported that nerve growth factor regulation of proliferation and the functionality of new born bovine testicular Sertoli cells (harvested, then treated in vitro) was through its receptor, *NTRK1*.Transferrin (*TF*) is a major transporter of iron into cells^31^ and was upregulated in the testes of bull calves in the HI versus MOD group in the current study. In the testis, where the blood-testis barrier prevents entry of macromolecules, iron is transported into the compartment from the circulatory system by transferrin produced in Sertoli cells^32^, potentially suggesting an increase in Sertoli cell production in the HI diet due to greater expression of *TF*. Indeed, English et al.^7^ reported a significantly greater number of Sertoli cells in the parenchyma of testes at 18 weeks of age and also an upregulated expression of *TF* in the same tissue. Furthermore, Fig. 2 highlights an interaction between these genes and androgen receptor (AR), further implicating DEGs in this study with testicular development. Indeed systemic concentrations of testosterone were greater in the HI calves used in the current study^10^ which may have led to the differential expression of genes within Fig. 2. Taken together, mRNA transcripts DE between calves on varying planes of nutrition during early life indicate towards greater reproductive and testicular development in the calves receiving the enhanced dietary intake.

Differentially expressed miRNAs of particular interest included miR-2419-5P and miR-11995 due the differential expression of their target mRNAs, *CDH13* and *TNFSF18*, respectively. An association between these miRNA and mRNA was established through the differential direction of effect, whereby each miRNA was up-regulated in the HI calves, whilst the corresponding target mRNA was down-regulated in the same dietary group, suggesting potential repression of mRNA transcription due to the actions of the respective miRNA. *CDH13* encodes a calcium-dependent cell adhesion, cadherin protein, and cadherin proteins have been shown to contribute to the association between Sertoli cells and spermatogenic cells in the testis^33^. Additionally, a recent co-expression network analysis undertaken by our group identified a negative association between a network containing CDH13 and the greater seminiferous tubule lumen scores evident in the testes of bull calves fed an enhanced diet up to 18 weeks of age^7^; indicating a down-regulation of this gene is associated with advanced testicular development in early life. *TNFSF18* is a member of the tumour necrosis factor family of cytokines which have important roles in normal testicular homeostasis^34^. Cytokines including TNF, are produced by the Sertoli or spermatogenic cells in a cyclical manner throughout the maturation cycles of the seminiferous epithelium. These actions suggest that they are key to regulating fundamental testicular functions^35^. Furthermore, TNF proteins also function as signalling molecules to regulate Sertoli cell function and cell death in response to toxic insults, and this activity is largely determined by the receptor with which it interacts^36^.

A secondary objective of this study was to integrate the ‘omics’ datasets generated. This was achieved through evaluating the relationship between DE-miRNA and DE-mRNA, as well as conducting a co-regulatory network analysis of proteomic data to determine interactions amongst the testes proteome profile. From the 5665 proteins identified in the testes parenchyma, a total of 13 co-regulated networks were derived. Of particular interest was the turquoise network, as this network contained the protein CDH13, the transcript of which, as mentioned above, was also observed to be directly related to miR-2419-5p. Pathway analysis of the turquoise network revealed enrichment of proteins with functions related to cholesterol biosynthesis, IGF-1 signalling, androgen signalling and Sertoli cell junction signalling. An interesting protein regulating CDH13 abundance was transferrin receptor (TFRC) given the role of *TF* in viability of Sertoli cells. Moreover, TFRC was observed to be differentially expressed at the transcript level in similar studies from our group^7^ and others^9^. The following pathways were also enriched from proteins interacting with CDH13; sirtuin and mTOR signalling pathways, oxidative phosphorylation and cholesterol biosynthesis. This may suggest a potential role for CDH13 in mediating the effect of metabolic status on testicular development, apparent through enrichment of sirtuin signalling and cholesterol biosynthesis pathways, respectively. Interestingly, protein co-regulatory analysis identified a role for CDH13 in regulating NT5E, the generator of adenosine, which is expressed by testicular peritubular cells and was identified as up-regulated in the HI calves in the current study. Moreover, CDH13, identified as a hub protein within the turquoise network was regulating other proteins including EBP, which functions in cholesterol biosynthesis and was also identified as up-regulated in the testes of bull calves offered enhanced diet up to 18 weeks of age^7^. Thus, the effect of enhanced metabolic status on steroid and fatty acid synthesis and testicular development may be mediated through CDH13 expression. Proteins related to fatty acid synthesis and cholesterol biosynthesis that CDH13 was interacting with were also previously identified as differentially expressed at the transcript level in the testes data of English et al.^7^ (*AACS, ACLY, ACSS2*) and Johnson et al.^9^ (*TM7SF2*). Moreover, in our earlier evaluation of the metabolic control of enhanced early life nutrition, we observed a tendency for greater systemic concentrations of cholesterol in HI compared with MOD calves, consistent with the higher concentrations of testosterone in the same calves used for the current study^10^. Overall, the main findings from the protein co-regulatory network analysis suggests a role for the CDH13 protein towards processes related to energy production and metabolic homeostasis (sirtuin signalling pathway, TCA cycle) as well as cell growth and proliferation and cholesterol biosynthesis towards the advancement of testicular development as a consequence of enhanced nutritional intake. However further functional analyses are warranted to fully determine the role of CDH13 towards advanced testicular development.

## Conclusion

Enhanced metabolic status during early calfhood affected the global testicular transcriptome of bull calves coincident with the early transient gonadotrophin rise (12 weeks). Offering calves, a high plane of nutrition during early calfhood phase (2–12 weeks of age) resulted in differential expression of both miRNA and mRNA between HI and MOD dietary groups. One miRNA of particular interest that was upregulated in the HI group was miR-2419-5p; with its corresponding target mRNA CDH13 down-regulated in HI calves, indicating a relationship between the miRNA and corresponding mRNA. Moreover, protein co-regulatory analysis indicated towards a role for the CDH13 protein towards mediating enhanced metabolic status with cholesterol biosynthesis within the testes, ultimately leading to earlier testicular development as a consequence of prevailing dietary management. Further functional analyses are warranted to fully determine the contribution of CDH13 towards advanced testicular development as a consequence of enhanced early life nutrition. Moreover, these findings warrant further attention, following appropriate validation, as potential molecular biomarkers for precocious testicular development in calves.

## Supplementary Information

Supplementary Tables.

## Data Availability

The sequencing data underlying this article are available in NCBI’s Gene Expression Omnibus at [https://www.ncbi.nlm.nih.gov/geo/] and is publicly available and can be accessed with unique GEO ID [GSE194041] and [GSE194305]. Proteomics data generated in this analysis have been uploaded to the ProteomeXchange Consortium via the PRIDE [http://www.ebi.ac.uk/pride] partner repository^16^ with the data identifier; [PXD031403], it is publicly available and can be accessed via the following link https://www.ebi.ac.uk/pride/archive/projects/PXD031403.
